# Neural Correlates of Processing Passive Sentences

**DOI:** 10.3390/brainsci3031198

**Published:** 2013-08-02

**Authors:** Jennifer E. Mack, Aya Meltzer-Asscher, Elena Barbieri, Cynthia K. Thompson

**Affiliations:** 1 Department of Communication Sciences and Disorders, Center for the Neurobiology of Language, Northwestern University, Francis Searle Building, 2240 Campus Drive, Evanston, IL 60208, USA; E-Mails: jennifer-mack-0@northwestern.edu (J.E.M.); elena.barbieri83@gmail.com (E.B.); 2 Department of Linguistics, Tel Aviv University, Webb Building, Ramat Aviv, Tel Aviv 69978, Israel; E-Mail: ameltzer@post.tau.ac.il; 3 Sagol School of Neuroscience, Webb Building, Tel Aviv University, Ramat Aviv, Tel Aviv 69978, Israel; 4 Cognitive Neurology and Alzheimer’s Disease Center, Feinberg School of Medicine, Northwestern University, 320 E. Superior, Searle 11-453, Chicago, IL 60611, USA; 5 Department of Neurology, Feinberg School of Medicine, Northwestern University, Abbott Hall, 11th Floor, 710 North Lake Shore Drive, Chicago, IL 60611, USA

**Keywords:** fMRI, sentence processing, syntactic processing, thematic processing

## Abstract

Previous research has shown that comprehension of complex sentences involving *wh*-movement (e.g., object-relative clauses) elicits activation in the left inferior frontal gyrus (IFG) and left posterior temporal cortex. However, relatively little is known about the neural correlates of processing passive sentences, which differ from other complex sentences in terms of representation (*i.e*., noun phrase (NP)-movement) and processing (*i.e*., the time course of syntactic reanalysis). In the present study, 27 adults (14 younger and 13 older) listened to passive and active sentences and performed a sentence-picture verification task using functional Magnetic Resonance Imaging (fMRI). Passive sentences, relative to active sentences, elicited greater activation in bilateral IFG and left temporo-occipital regions. Participant age did not significantly affect patterns of activation. Consistent with previous research, activation in left temporo-occipital cortex likely reflects thematic reanalysis processes, whereas, activation in the left IFG supports processing of complex syntax (*i.e.*, NP-movement). Right IFG activation may reflect syntactic reanalysis processing demands associated with the sentence-picture verification task.

## 1. Introduction

A considerable body of research has investigated the neural basis of sentence comprehension by comparing complex to simple sentences. One dimension of sentence complexity is the presence *vs*. absence of *wh*-movement, which influences the mapping of verb arguments onto the surface representation of sentences. Compare a simple active sentence (1a) to an object *wh*-question (1b):

a. The boy is hugging the girl.
b. Who_i_ is the boy hugging t_i_?
c. The girl_i_ was hugged t_i_ by the boy.

Both sentences require the listener to build a syntactic structure that guides the integration of the verb *hug* with its arguments (*i.e*., event participants that receive a thematic role from the verb): the agent (performer of the action) and the theme (undergoer of the action). However, these processes are more difficult for (1b) than (1a). Some linguistic theories (e.g., Government and Binding Theory; [1]) claim that in object *wh*-questions, the theme argument originates in the post-verbal position, as it does in its active counterpart, and is displaced to the beginning of the sentence, leaving behind a “trace” of the movement operation, co-indexed with the moved *wh*-word. As a result, object *wh*-questions have a *noncanonical* verb-argument structure in which the theme precedes the agent, in contrast with *canonical* active sentences in which the agent is mapped to the subject position, preceding the theme, which is mapped to the object position. Thus, object *wh*-questions are more complex than simple active sentences. Previous psycholinguistic studies have found that the moved *wh*-word (the *filler*) is reactivated immediately after the verb (the *gap*, *i.e*., the hypothesized trace site), lending support to the psychological reality of *wh*-movement [2,3,4,5,6,7]. For example, using a visual world paradigm, *wh*-movement structures (including object-extracted *wh*-questions and object cleft structures) elicit automatic eye movements to the filler at the gap site in healthy adults and listeners with aphasia [2,3]. Additional evidence comes from event-related potential (ERP) studies [8], indicating that *wh*-embedded questions—as compared to *whether*-questions that do not entail gap-filling—elicit a positivity at the gap position around 400–700 ms.

Passive sentences (1c) also are noncanonical and, hence, are more complex than canonical, active forms (1a). In passive sentences, the theme is the grammatical subject, and the agent is mapped to an adjunct prepositional phrase. According to some theorists, passive sentences also involve syntactic movement [1], *i.e*., NP-movement (due to the movement of a noun phrase), in contrast with *wh*-movement, in which a *wh-*word is displaced. In movement-based accounts of passive sentences, the theme originates post-verbally in the direct object position and moves to the grammatical subject position. However, in other linguistic theories (e.g., Head-Driven Phrase Structure Grammar [9] and Lexical-Functional Grammar [10]), passive sentences do not involve movement; instead, passive sentences are distinguished from actives solely on the basis of lexical/thematic structure. 

These accounts make different predictions about the processing of passive sentences. Movement-based accounts predict that identification of a passive structure during language comprehension triggers *syntactic reanalysis*, in which a trace is constructed and co-indexed with the grammatical subject, whereas lexical/thematic accounts do not. Psycholinguistic studies have found that passive sentences elicit delayed or absent reactivation of the filler at the hypothesized gap site, in contrast with *wh*-movement structures. One cross-modal priming study [11] found evidence of delayed reactivation of the grammatical subject, with effects reaching significance only 1000 ms after the verb. Similarly, delayed reactivation effects have been reported for unaccusative structures (e.g., *The leaf_i_ fellt_i_*) that are also hypothesized to involve NP-movement [12,13,14]. These findings are consistent with the claim that syntactic reanalysis does take place in structures with NP-movement, though on a slowed time course. Other studies, however, have failed to show gap-filling effects for passive structures with either healthy or aphasic listeners either at the gap site or downstream from it [3]. Thus, it remains an open question whether passive sentences require syntactic reanalysis. 

Both accounts predict that passive sentences, like other noncanonical sentences, should trigger a process of *thematic reanalysis*, *i.e*., revision of an initial mapping of thematic roles. Psycholinguistic studies have shown that healthy listeners tend to interpret sentence-initial noun phrases as agents unless there are additional cues to thematic mapping in the linguistic representation, such as case-marking [15], or in the discourse context [16]. This “agent-first bias” has been demonstrated through visual-world eye tracking studies in which listeners tend to direct initial looks to scenes in which the first noun phrase is the agent ([15,17,18,19,20], although *cf.* [21]). In the case of active (canonical) sentences, the agent-first bias corresponds to the true structure of the sentence, and no reanalysis is required. However, passive (noncanonical) sentences require thematic reanalysis as soon as the structure is identified as passive (in English, upon encountering the past participial morphology characteristic of the passive voice). At this point, the thematic role assignment of the first noun phrase must be revised from agent to theme. Some studies have found longer reaction times for passive as compared to active sentences [22,23], which may be due to the processing costs of thematic reanalysis and/or syntactic reanalysis. 

Studies examining the neural mechanisms of sentence processing have primarily focused on *wh*-movement structures by comparing neural activation patterns elicited by complex *versus* simple sentences, such as object and subject relative structures [24,25,26,27,28]. These studies consistently report activation in left inferior frontal gyrus (IFG) (see [29], for review), with some researchers suggesting that this activation reflects syntactic movement operations [30,31,32,33,34]. Although few neuroimaging studies examining NP-movement structures have been reported, all studies contrasting passive and active sentences have also found activation in the left IFG ([26,35,36,37,38]; *cf.* [39], who report activation in the left frontal operculum). Shetreet, Friedmann and Hadar [34] also found left IFG activation in an fMRI study of Hebrew unaccusative sentences, which like English passive structures, involve NP-movement (but, see [40], who did not find IFG activation for a different NP-movement structure in German). Notably, the majority of passive sentence processing studies have been conducted with Japanese-speaking participants and, as pointed out by Yokoyama *et al*. [37], in Japanese, passive verbs are morphologically marked (*i.e*., by the morpheme *rare*), whereas active verbs are uninflected. Thus, the IFG activation for passive sentences may be attributable to morphological complexity, rather than movement operations. Japanese also is a verb-final language and, therefore, the thematic and syntactic reanalysis processes take place after both noun phrases are presented (see [38] for discussion). In addition, Japanese has three types of passive sentences [41]. For these reason, it is an open question whether English passive sentences elicit patterns like those for Japanese passives.

Several neuroimaging studies of complex *wh*-movement sentences also have reported left posterior perisylvian activation; however, fewer NP-movement structure studies find this pattern (see [35,36,37,38], who reported no posterior activation). These regions include the posterior middle and superior temporal gyri (pMTG, pSTG) and the inferior parietal cortex, (*i.e*., angular gyrus (AG)). Notably, these regions have been found to reflect verb-argument structure processing [42,43,44], as well as the integration of verbs with their arguments ([45,46]; see [47] for review). These findings suggest that left posterior perisylvian regions may support thematic reanalysis, but this raises the question of why activation in these regions has not emerged consistently for passive sentences. One possible explanation is that task demands influence the likelihood of observing activation in this region, with activation more likely when the task places significant demands on thematic reanalysis processes. 

Hirotani *et al*. [26] aimed to disentangle the neural correlates of thematic and syntactic reanalysis by comparing active sentences (which require neither thematic nor syntactic reanalysis), passive sentences (which are assumed by the authors to require both) and causative sentences (which are claimed to require thematic reanalysis, but not syntactic reanalysis, because they do not involve syntactic movement). Relative to active sentences, both passive and causative sentences elicited activation in regions, including the left IFG (pars triangularis) and the left posterior superior temporal gyrus (pSTG), whereas direct comparison of passive to causative sentences showed no differential activation. However, the time course of activation differed for the two sentence types in the left IFG (greater activation for passive than causative sentences approximately 8 s after the critical point of the sentence), but not in the pSTG. The authors argue that both the left IFG and pSTG support thematic reanalysis, whereas the left IFG additionally supports syntactic reanalysis. 

Studies of aphasia also provide insight into the neural basis of complex sentence processing. Individuals with agrammatic (Broca’s) aphasia, resulting from stroke or head-injury, typically have impaired production and comprehension of noncanonical sentences, including both *wh*- and NP-movement structures [48,49,50,51]. Because agrammatic aphasia often is associated with damage to Broca’s area, this pattern suggests that Broca’s area plays a crucial role in complex sentence processing. Other research, however, shows that stroke-induced lesions in agrammatic aphasia often extend well beyond Broca’s area and include cortical (and subcortical) tissue in temporoparietal regions, as well (see [52,53], for lesions associated with stroke-induced agrammatic aphasia). In addition, studies directly investigating the relationship between complex sentence processing and lesion location have reported mixed results: damage to anterior and/or posterior perisylvian regions can result in deficits in complex sentence processing [54,55,56]. Two recent studies examining cortical atrophy in patients with the agrammatic variant of primary progressive aphasia (PPA-G) found correlations between cortical atrophy in the left IFG and impaired complex sentence processing in these patients [57,58]. However, Caplan and colleagues [55] argued for a primary role of posterior perisylvian regions in syntactic processing in stroke-induced aphasia. The authors found that stroke-induced lesions in both Wernicke’s area, and the anterior inferior temporal lobe were associated with accuracy on a sentence-to-picture matching task, whereas lesions in the inferior and superior parietal lobe predicted performance on a task tapping thematic role assignment and co-indexation for syntactically complex sentences, as compared to simple sentences. In addition, Thothathiri, Kimberg, and Schwartz [59], in a voxel-based lesion mapping (VLSM) study in stroke-induced aphasia, found correlations between noncanonical sentence comprehension deficits and lesions in the left temporo-parietal cortex, as well as other regions, but not in Broca’s area. These mixed results may be due in part to variable deficits in thematic and/or syntactic reanalysis processes across individuals with aphasia. 

Collectively, the results of both neuroimaging studies with healthy participants and lesion-deficit correlation studies suggest that both anterior and posterior regions are engaged for complex sentence processing. However, because of the dearth of studies examining NP-movement structures in English, as well as the mixed findings derived from Japanese studies focused on these sentences, further research is needed. In addition, further evidence is necessary to test the hypothesis that the left IFG supports syntactic reanalysis whereas, left posterior perisylvian regions support thematic reanalysis during complex sentence processing (*cf.* [26]). 

The present study investigated the neural correlates of processing passive and active sentences in English, using a sentence-picture verification task to probe comprehension. This task was selected in order to maximize demands on thematic mapping and reanalysis processes by requiring the participant to integrate the linguistic representation with an external representation of the event (the visual scene). Following previous studies, we expected to find activation in the left IFG for passive as compared to active sentences. Despite mixed findings with respect to left posterior perisylvian activation, we expected activation in these regions, as well, due to our use of a thematically-demanding task. If passive sentences entail syntactic reanalysis, as suggested based on linguistic descriptions of passives as NP-movement structures, we expected greater IFG activation for passive as compared to active sentences. Additionally, if passive sentence computation engages thematic reanalysis, posterior perisylvian activation would be expected. 

## 2. Results and Discussion

### 2.1. Behavioral Results

Participants performed overall very well on both active (97.7%) and passive (98%) sentences. Accuracy was equally good when the sentence matched the picture displayed on the screen and when sentence and picture did not match (98.1% and 97.5%, respectively). Reaction times (RT) obtained on correct trials were faster for active sentences (3519 ± 362 ms) as compared to passive sentences (3578 ± 375 ms) and for matched as compared to mismatched sentences (3497 ± 335, 3601 ± 395). A mixed-effects regression analysis was conducted by introducing one predictor at a time: *sentence type* (active, passive), *condition* (match, mismatch) and *age*, to evaluate the contribution of each to the variance. ANOVAs between consecutive models were run with subjects and items introduced as random factors for all. Random slopes for significant predictors were also introduced in the model one at a time, and their contribution to the explanation of variance was evaluated in the same way as the fixed factors. Prior to the analysis, reaction times were log-transformed in order to render a normal distribution. Results indicated a significant main effect of sentence type (*t* = 2.2, *p* = 0.031), with longer RT for passive as compared to active sentences and of condition, where sentences that did not match the picture elicited longer RT than sentences matching the picture (*t* = 3.7, *p* < 0.001). The introduction of random slopes for both sentence type and condition significantly improved the model (*χ*^2^ = 6.8, *p* = 0.033 and *χ*^2^ = 26.4, *p* < 0.001 respectively). No significant interaction between sentence type and condition emerged (*t* = −0.4, *p* = ns). *Age* did not reach significance as a predictor (*t* = 1.15, *p* = ns). 

### 2.2. fMRI Results

**Figure 1:**
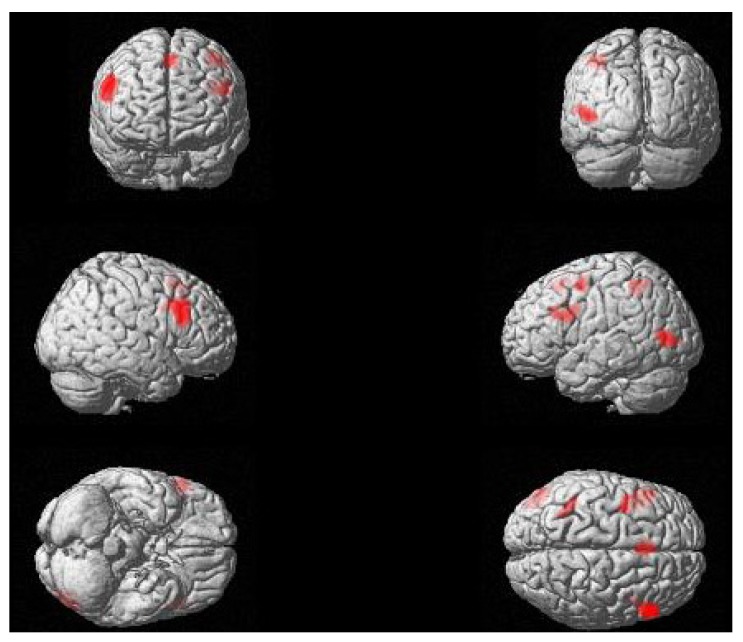
Regions of differential activation for passive as compared to active sentences (voxel-wise threshold of *p* < 0.001; *k* ≥ 85).

Passive sentences, as compared to active sentences, elicited significant clusters of activation in the bilateral inferior frontal gyrus (pars opercularis and pars triangularis, overlapping with BA’s 44 and 45), as well as in the left temporo-occipital junction (middle occipital gyrus and posterior middle temporal gyrus); see Table 1 and Figure 1. Additional clusters that did not survive correction for multiple comparisons were observed in the bilateral supplementary motor area, the left superior parietal lobule, and the left precentral gyrus. The reverse contrast, of active over passive sentences, did not reveal any significant areas of activation. In addition, no significant effects of age were observed for either contrast (passive > active, active > passive). 

**Table 1:** Areas of differential activation for passive and active sentences. Peak Montreal Neurological Institute (MNI) coordinates, cluster sizes (*k*), maximal *t-*values, and cluster-corrected (family-wise error rate) *p-*values are reported (voxel-wise threshold of *p <* 0.001, uncorrected, *k* ≥ 85). Notes: LH, left hemisphere; RH, right hemisphere; IFG, inferior frontal gyrus; pMTG, posterior middle temporal gyrus; SMA, supplementary motor area; SPL, superior parietal lobule.

Contrast	Region	Peak Coordinates			*k*	*t*	*p*
		*x*	*y*	*z*			
Passive > Active	RH IFG (pars opercularis, pars triangularis)	56	24	28	497	6.09	0.005
	LH IFG (pars opercularis, pars triangularis)	−36	4	32	349	4.52	0.019
	LH middle occipital gyrus, pMTG	−46	−76	4	265	5.21	0.048
	Bilateral SMA	−2	22	54	217	4.77	0.084
	L SPL	−40	−46	52	136	3.60	0.227
	L precentral gyrus	−38	−2	54	117	4.2	0.289
Active > Passive Age	*None*						
(Passive > Active, Active > Passive)	*None*						

### 2.3. Discussion

The present study investigated the neural correlates of processing passive sentences in English. Consistent with previous studies, we found activation for passive sentences in the left IFG, including tissue in both the pars opercularis and pars triangularis (*cf.* [26,36,37,38,39]); additionally, activation was found in homologous regions in the right hemisphere. We also found activation in the left temporo-occipital junction, including the pMTG. As pointed out earlier, some previous studies have reported posterior perisylvian activation for passive as compared to active sentences [26,39], whereas others have not found activation in this region [36,37,38]. These results contribute to our understanding of the neural basis of complex sentence processing, in particular, the functions of the left and right IFG and left posterior temporal regions in supporting noncanonical sentence comprehension. 

#### 2.3.1. Roles of the Left and Right IFG in Passive Sentence Comprehension

Several explanations have been proposed for the observation that complex sentence comprehension consistently elicits activation in the left IFG. It has been argued by some researchers that the left IFG supports the processing of complex syntactic representations [24,25,53,60,61,62] or, more specifically, syntactic movement [30,31,32,33,34], whereas other accounts suggest that the IFG supports morphological processing [37], semantic/pragmatic aspects of argument processing [63,64,65,66,67] or working memory [68]. Results of the present study are not consistent with morphological, semantic or working memory accounts of left IFG activation. First, the experimental sentences were controlled for morphological complexity, through the use of auxiliary verbs combined with present participles in active sentences and past participles in passive sentences. Second, the experimental sentences were semantically reversible, with the same arguments used in passive and active sentences, and therefore, left IFG activation is not likely due to processing the intrinsic semantic features of arguments. Third, working memory demands were low in the present study, due to the use of short sentences; furthermore, in contrast with *wh-*structures, passive sentences are unlikely to place enhanced demands on working memory, because the subject NP is not identifiable as a filler until the verb is processed. Thus, the IFG activation found here is most likely associated with the greater syntactic and/or verb-argument structure complexity of passive as compared to active sentences. The present results, however, do not directly address whether the source of left IFG activation is syntactic movement and/or noncanonical verb-argument structure mapping. Previous research suggests that the left IFG is involved in both aspects of syntactic complexity, supporting both thematic and syntactic reanalysis processes [26]. Studies also show that these processes may be supported by different subregions of the IFG, although research examining the neural substrates of syntactic movement report peak activation in the pars opercularis (roughly BA 44) [25,27], as well as in the pars triangularis (roughly BA 45) [26,35,36,38]; see discussion in [29]. Similarly, both the pars opercularis and pars triangularis have been linked to noncanonical argument mapping (see discussion in [64]). 

Thus, the present results are consistent with the view that the left IFG supports syntactic movement [30,31,32,33,34]. However, they are incompatible with accounts claiming that the IFG supports only certain types of movement: that is, *wh*-movement, but not NP-movement found in passive sentences. For example, Christensen [69] argues that movement to the complementizer phrase (CP) domain (*i.e*., movement to the left of the grammatical subject position), which takes place in *wh*-movement structures, should elicit activation in the left IFG and posterior perisylvian regions, whereas movement within the inflectional phrase (IP) domain (*i.e*., movement to or to the right of the grammatical subject position), which occurs in NP-movement structures, such as passive sentences (under standard assumptions), should elicit activation in the left anterior temporal cortex instead. Santi and Grodzinsky [28] propose that left IFG activation is elicited only by movement that is predictable at the point of the antecedent (*i.e*., *wh*-movement structures in which the *wh*-word is immediately identifiable as a filler). However, consistent with previous studies on passive sentence processing in Japanese and Chinese [26,35,36,37,38,39], our findings suggest that if indeed, the left IFG supports syntactic movement; it does so not only for *wh*-movement structures, but also for NP-movement structures. Furthermore, despite differences between Japanese and English passives with respect to linguistic representation [41] and processing costs [39], the present results indicate that English passives, like Japanese passives, elicit left IFG activation, possibly because passive sentences in both languages require syntactic reanalysis. 

In addition, passive sentences elicited greater activation in the right IFG. This finding contrasts with previous studies of passive sentence comprehension, which did not find activation in the right IFG ([26,35,36,37,38,39]), and in general, effects of syntactic complexity in the IFG tend to be strongly left-lateralized (for a review, see [29]). However, some evidence suggests that right IFG supports syntactic reanalysis of complex sentences, particularly in the context of integration of a linguistic representation with a visual scene. In an fMRI study, Meltzer, McArdle, Schafer and Braun [62] investigated the neural correlates of syntactic reanalysis demands during comprehension of complex (noncanonical object-relative) and simple (canonical subject-relative) sentences. In 50% of the trials, participants simply listened to sentences (sentence-alone condition); in the other 50% of the trials, participants performed a sentence-picture matching task. Syntactic reanalysis demands were higher in the sentence-picture matching condition, because participants were required to hold the linguistic representation of the sentence in memory, compare it to the picture probes, then reanalyze it, if necessary. The authors reported main effects of syntactic complexity (object-relatives > subject-relatives) in the left IFG across tasks; however, in sentence-picture matching trials, effects of syntactic complexity were observed in bilateral IFG. The authors propose that the right IFG specifically supports conscious and effortful syntactic reanalysis. Similarly, right IFG activation has been found in previous studies that required participants to generate linguistic judgments about syntactic structures [70,71]. The present study also placed high demands on syntactic reanalysis processes, as participants were required to build a linguistic representation of the sentence and compare it to a visual scene. Thus, high syntactic reanalysis demands are one plausible explanation for the right IFG activation observed for passive sentences. However, we note that the design of the present study (passive/active blocks) makes it difficult to distinguish the neural correlates of reanalysis processes that are due to sentence type (passive *vs*. active sentences) from those that are due to the correspondence between the sentence and picture (mismatch *vs*. match trials).

A less likely explanation for the presence of bilateral IFG activation is the inclusion of both young and older adult participants. Some previous research has suggested that healthy aging is associated with increased bilateral language activation, especially in frontal regions [72,73,74]. Therefore, one might hypothesize that bilateral IFG activation for passive sentences in the present study was driven by the older participants. However, we did not observe any significant effects of age on the processing of passive (or active) sentences. For this reason, it is more likely that bilateral IFG activation is due to the high syntactic reanalysis demands imposed by the sentence-picture verification task. 

#### 2.3.2. The Role of Left Posterior Temporal Cortex in Passive Sentence Comprehension

Previous research has shown that left posterior temporal cortex supports thematic reanalysis in passive sentences [26], and more generally, the integration of verbs with their arguments [29,43,44,45,47]. Therefore, the left temporo-occipital activation observed for passive sentences in the present study is likely due to thematic reanalysis in the context of a sentence-picture verification task. However, it is an open question why previous studies of passive sentence processing have yielded mixed results with respect to activation in this region. One possible explanation is that posterior perisylvian activation is more sensitive to the choice of task than is left IFG activation, such that tasks that place greater demands on thematic mapping and reanalysis processes are more likely to elicit posterior perisylvian activation. Consistent with this hypothesis, Caplan, Chen and Waters [61] found left IFG activation for noncanonical as compared to canonical sentence comprehension across three tasks (sentence verification, plausibility judgment and non-word detection), whereas posterior perisylvian activation was found only for the sentence verification and plausibility judgment tasks, both of which target thematic role mapping. Studies of passive sentence processing have reported nonhomogeneous findings that nonetheless provide some support for this hypothesis. For example, Yokoyama *et al*. [37] used a lexical decision task that did not target thematic role mapping and did not elicit posterior perisylvian activation. Studies that targeted thematic role mapping within the sentence (e.g., plausibility judgments, comprehension questions about thematic role assignment) have yielded mixed results, with some studies [26,39] eliciting temporo-parietal activations and others not [36,38]. In addition, one study [35] used a sentence-picture verification task, as in the present study. The authors did not find posterior perisylvian activation for passive as compared to active sentences, but did find posterior temporal (pMTG/pSTG) activation for another type of noncanonical structure (scrambled sentences) as compared to active sentences. Thus, there is some evidence that activation for passive sentences may emerge in left posterior perisylvian regions only with tasks that place explicit demands on verb-argument integration. 

The left posterior temporo-occipital activation found in the present study is located inferiorly and posteriorly to the pSTG activation reported by Hirotani *et al*. [26] and in studies of verb-argument integration [29,47]. However, we note that some studies of complex sentence processing have elicited activation in the left temporo-occipital junction [62,75,76]. Meltzer and colleagues [62] also found left pMTG/pSTG activation for semantically reversible relative to non-reversible sentences in both sentence-alone and sentence-picture matching conditions. However, this activation was shifted posteriorly in the sentence-picture matching condition (peak MNI coordinates: (−49 −73 4); *cf.* peak coordinates in the present study: (−46 −76 4)) relative to the sentence-alone condition (peak coordinates: (−53 −46 11)). This suggests that integration of auditorily-presented linguistic stimuli with visually-presented scenes may result in a shift posteriorly to the temporo-occipital junction for thematic processing (see, also, the discussion of task effects on activation patterns in [29]).

## 3. Experimental Section

### 3.1. Participants

Fourteen healthy young adults (mean age: 24.9; range = 19–38; two males) and thirteen healthy older adults (mean age: 61.2; range = 54–70; seven males) participated in the study. All were right-handed native speakers of English with normal vision and hearing and no history of speech/language, learning or neurological disorders. The study was approved by the Institutional Review Board at Northwestern University, and all participants gave informed consent.

### 3.2. Materials

Twenty verbs were selected for inclusion in the experiment. All were semantically reversible, frequently-occurring (*M* log frequency = 4.33; Corpus of Contemporary American English (COCA); [77]) and had a regular passive form (-*ed*). Each verb was embedded in four sentences, all including the same noun phrase participants: two active sentences (e.g., *The brother was pushing the sister*; *The sister was pushing the brother*) and two passive sentences (e.g., *The brother was pushed by the sister*; *The sister was pushed by the brother*). All sentences contained past-tense verb forms; the passive sentences included a past-tense auxiliary combined with the past participle (*was V*-*ed*), whereas the active sentences contained a past-tense auxiliary with a progressive main verb (*was V-ing*), in order to control for morphological complexity across the two conditions. The two sentence types were also controlled for length in syllables (active *M* = 6.15; passive *M* = 6.3, *p* =0.42). The nouns referring to participants were all frequently-occurring (*M* log frequency = 5.00, COCA) and referred to humans or animals (e.g., *brother, cat, mouse, woman*)***.***

Sentences were recorded by a female native English speaker in a sound proof booth, using Audacity. Maximum amplitude of the sound files was normalized to −3 dB. All sentences were between 2.5 and 3 s, with 100 ms of silence added at the offset of each sentence. An additional variable period of silence was added at the beginning of each sentence, so that all sound files were 3500 ms long. 

Twenty black and white drawings were prepared, one for each verb. All drawings depicted two animate participants engaged in an event, such that the agent acted upon the theme (see, e.g., Figure 1). The same line drawing was used for all four sentences constructed with the same verb.

### 3.3. Procedures

In each trial, a picture and a sentence were presented simultaneously, with the picture remaining on the screen for 6000 ms, followed by 1000 ms fixation. Participants held a response box in their left hand and were asked to press with their index finger if the picture matched the sentence and with their middle finger if it did not. Participants could respond at any point during the trial, and reaction times were measured relative to the onset of stimulus presentation. The experiment was presented using E-Prime, and participant responses were recorded using Cedrus RB610 response boxes.

The experiment used a block design, such that each block included four trials of the same syntactic structure (active or passive), with matched and mismatched trials pseudorandomized within and across blocks. Each block ended with a 12 s fixation cross, for a total of 40 s per block. The experiment included 20 blocks, which were pseudorandomized and assigned to two runs of 6:40 min each. The order of presentation of the two blocks was counterbalanced across participants.

### 3.4. Data Acquisition

MRI data were acquired using a Siemens 3T Tim Trio scanner with a 32-channel head coil. At the beginning of each scan, a T1-weighted anatomical image was acquired, with the following parameters: time to repeat (TR) = 2300 ms, time to echo (TE) = 2.91 ms, flip angle = 9 degrees; matrix size = 256 × 256; field of view (FOV) = 256 mm; voxel size = 1 × 1 × 1 mm; 176 slices). During the experimental task, blood oxygen level dependent (BOLD) contrast images were acquired using the following parameters: TR= 2000 ms; TE = 30 ms, flip angle = 80 degrees; matrix size = 64 × 64; FOV = 220.16 mm; voxel size = 3.44 × 3.44 × 3 mm; 32 slices. 

### 3.5. Data Analysis

#### 3.5.1. Behavioral Data

Accuracy and reaction time (RT) data were analyzed by means of logistic (for accuracy) or linear (for RT) regression with mixed-effects, following the approach described by Jaeger [78] and Baayen, Davidson and Bates [79]. Subject and item were introduced as random effects in the regression analysis, and the contribution of each predictor to the explanation of the variance was evaluated by performing ANOVA comparisons between models. 

#### 3.5.2. Neuroimaging Data

Preprocessing and statistical analysis of the MRI data were performed using SPM8. Preprocessing consisted of slice-timing correction of the functional scans, realignment of the functional scans to a mean functional volume, normalization of the anatomical and functional scans to the MNI 152-subject template brain, reslicing of functional and anatomical scans to a 2 × 2 × 2 mm voxel and smoothing of the functional images using a 9 mm Gaussian kernel.

In order to eliminate scanner drift, a high-pass filter of 128 s was used in the first-level statistical analysis. In addition to the two experimental conditions (active, passive), a parameter for run and six motion parameters were entered into this analysis. In second-level analyses, contrast maps from each participant (passive > active sentences; active > passive sentences) were entered into ANCOVA analyses. Participant age was entered as a covariate to control for and evaluate the potential effects of age on passive and active sentence processing. The results were evaluated using a voxel-level threshold of *p* < 0.001 with a minimum cluster size (*k*) of 85 (680 mm^3^), applying family-wise error rate correction at the cluster level to identify clusters of significant activation. This is equivalent to, or more stringent than, the threshold used in some previous studies testing subtle linguistic contrasts (e.g., [42] and references therein). 

## 4. Conclusions

The results of the present study suggest that in both younger and older adults, comprehension of passive sentences is supported by bilateral IFG and left posterior temporo-occipital regions. These findings are largely consistent with previous research on the comprehension of complex sentences, which has linked left IFG activation to syntactic complexity and left posterior temporal activation to verb-argument structure integration (see, e.g., [29,80]). Thus, despite the linguistic and psycholinguistic differences between passive sentences and other complex structures, they may be largely supported by the same brain regions. The right IFG activation found in the present study may reflect effortful reanalysis processes required by the sentence-picture verification task. Together with previous studies [62,70,71], this suggests that the right IFG may play a role in controlled syntactic reanalysis and the generation of linguistic judgments.
